# Complementary white matter networks support flexible concept learning

**DOI:** 10.3389/fnins.2026.1877268

**Published:** 2026-09-30

**Authors:** Melisa Gumus, Nahal Alizadeh Saghati, Michael L. Mack

**Affiliations:** 1 Department of Psychology, University of Toronto, Toronto, ON, Canada; 2 Department of Psychology, University of California Berkeley, Berkeley, CA, United States

**Keywords:** brain networks, category learning, correlational tractography, diffusion imaging, white matter

## Abstract

**Introduction:**

Concept learning is not an isolated act of cognition; it recruits a host of core mechanisms, including working memory, decision making, and attention. A wide range of brain networks spanning cortical and subcortical areas support these operations, which have been identified via activation patterns related to concept learning. A critical open question, however, is whether the white matter architecture connecting these regions serves as the structural scaffold that makes such flexible learning possible.

**Methods:**

We employed a rule-plus-exception learning paradigm wherein participants categorized visually complex stimuli. While some items closely followed the rules, exceptions violated them, acting as a behavioural index of flexible learning. We quantified the integrity of white matter tracts across the whole human brain using diffusion-weighted imaging and leveraged correlational tractography to uncover their behavioural relevance.

**Results:**

Correlational tractography revealed two distinct white matter networks associated with individual variability in either rule or exception learning. The network supporting exception learning was predominantly subcortical-limbic, comprising the fornix, thalamic radiations, and forceps minor alongside the left frontal-parietal cingulum, a pattern consistent with hippocampal-prefrontal communication and cognitive control over atypical exemplars. Conversely, the network for rule-based items was distinguished by long-range cortico-cortical and cortico-cerebellar pathways, including the right frontal-parietal cingulum, the middle longitudinal fasciculus, and the middle cerebellar peduncle, reflecting the integration of visual features with abstract rule representations and their gradual automatization.

**Discussion:**

These findings provide novel evidence that complementary white matter networks support flexible concept formation and offer a neuroanatomical basis for characterizing individual differences in flexible concept learning.

## 1 Introduction

Concept learning is a fundamental cognitive process of organizing information into structured knowledge. Research into the neural signatures of concept formation underscores that concept learning is not a monolithic process executed by a single brain region, but rather that it is supported by distributed brain networks. Acquiring new concepts relies on multiple cognitive mechanisms: processing visual information, tuning attention to relevant features, developing organizational strategies in working memory, and determining the class of newly encountered information (Ashby and Maddox, 2005; Rehder and Hoffman, 2005; Zeithamova and Maddox, 2007; Kumaran et al., 2009; Seger and Miller, 2010; Mack et al., 2016; Zeithamova et al., 2019). Consequently, the learning process recruits diverse neural systems, including the frontal and parietal regions, medial temporal lobe, and subcortical regions (Ashby et al., 1998; Martin, 2007; Seger and Miller, 2010). While extensive research has illuminated the functional properties of these distinct brain regions in concept learning, the role of white matter connections between such brain regions remains largely unexplored. As these structural connections are crucial for facilitating neural communication across the brain (Scholz et al., 2009), understanding their contribution is essential for revealing the dynamic interactions between widespread brain networks during concept learning. We address this gap by quantifying whole-brain white matter connections in healthy young adults, thereby identifying a set of white matter networks that are integral to flexible concept formation. Specifically, we focus on two types of concept learning that place distinct demands on the cognitive system: learning rule-following items, which conform to the defining features of a category, and learning exceptions, which violate those rules and must be encoded as distinct exemplars despite their surface similarity to members of the opposing category.

The distributed brain network underlying concept learning likely involves a cascade of information processing across various brain regions: perceptual cortices such as early visual and lateral occipital cortex are crucial for initial complex object feature encoding (Malach et al., 1995), the posterior parietal areas for directing attention to relevant dimensions (Corbetta and Shulman, 2002), the hippocampus for binding features into episodic exemplars and initiating generalization (Mack et al., 2018), and the prefrontal regions for integrating information into schemas and representing more abstract prototypes (Gilboa and Marlatte, 2017; Bowman and Zeithamova, 2018). Information first arrives in the visual cortex before being organized into concepts in higher-order cortical regions (Ashby and Maddox, 2011; Richler and Palmeri, 2014). Acquired information is transformed according to its perceptual similarities in a psychological space, which is often reflected in the neural responses of the visual cortex (Folstein et al., 2013). Visual information is then integrated into perception by the parietal cortex to generate a behaviourally relevant response (Seger and Miller, 2010). The posterior parietal cortex, in particular, has received much attention in categorization tasks because of its unique connections between the visual areas and higher cortical regions (Fitzgerald et al., 2012). Accumulating evidence suggests that the posterior parietal cortex houses conceptual information such as item-specific, concept-related information, and category rules (Freedman and Assad, 2006; Swaminathan and Freedman, 2012; Kuhl and Chun, 2014) although, traditionally, the ventral temporal cortex has been considered the main region for reflecting conceptual knowledge and category selectivity (Hoffman and Lambon Ralph, 2018). Beyond the parietal regions, category representations have also been identified in the prefrontal cortex, highlighting the complex interplay between these interconnected areas.

Selective attention, a process heavily modulated by the prefrontal cortex, has been considered a crucial part of concept formation in computational models (Shepard et al., 1961). Attention highlights task-relevant features and filters out irrelevant information (Nosofsky, 1986; Kruschke, 1992), configuring the concept space for learned information (Corbetta et al., 1991; Love et al., 2004; Rehder and Hoffman, 2005; Mack et al., 2020; Luo et al., 2021). This attention optimization creates new concepts in long-term memory or updates existing ones, contributing to the flexibility of the categorization process (Mack et al., 2026). These learned concepts, including task contingencies, concept members, and task rules, have been shown to be stored for the long-term in the prefrontal cortex (Miller and Cohen, 2001; Miller et al., 2002; Seger and Miller, 2010; Constantinescu et al., 2016). Neurobiological evidence from primate studies emphasizes the importance of the lateral portion of the prefrontal cortex in encoding category structures, enabling executive processing, and a behavioral response to stimuli (Freedman et al., 2002, 2003). Further evidence suggests a regional distinction in the lateral prefrontal cortex such that the ventrolateral prefrontal cortex is engaged more in concrete categories while the dorsolateral prefrontal cortex is reported to be involved in higher-level abstraction of concepts (Wutz et al., 2018). In sum, the prefrontal cortex acts as a key top-down attention processor, optimizing attention to task-relevant content during learning (Mante et al., 2013) while its ventromedial portion compresses the complexity of the learned information at the end of learning (Mack et al., 2020). Thus, the prefrontal cortex is critical in building knowledge, yet it does not act alone.

The hippocampus, specifically its anterior portion, couples with the prefrontal cortex during learning while concepts are being created (Mack et al., 2016). The coupling between the prefrontal cortex and hippocampus enables the integration of new memory traces into existing knowledge (Schlichting and Preston, 2017) and the generalization of learned concepts to novel situations (Bowman and Zeithamova, 2018; Frank et al., 2019; Zeithamova and Bowman, 2020). While the hippocampus has traditionally been evaluated with respect to its role in episodic encoding and retrieval, recent findings suggest that its role expands into concept learning (Hutter and Wilson, 2018; Mack et al., 2018). Notably, the hippocampal functions such as pattern separation or completion, as well as extracting regularities over experiences (O’Reilly and Rudy, 2001; Norman and O’Reilly, 2003; Schlichting and Preston, 2015; Schapiro et al., 2017), are critical for concept learning (Davis et al., 2012a; Mack et al., 2016, 2018; Bowman and Zeithamova, 2018; Zeithamova et al., 2019). Moreover, hippocampal representations reflect newly acquired knowledge, making it a candidate region for transforming episodic experiences into organized concepts (Love and Gureckis, 2007; Aly and Turk-Browne, 2016; Mack et al., 2018). While flexible knowledge formation is often supported by the medial temporal lobe, a more automated and procedural learning process is traditionally believed to involve the striatum (Poldrack et al., 2001), although it can also engage in declarative processes depending on the task context (Foerde et al., 2006). Rather than focusing on traditional dissociations between declarative and procedural systems, modern approaches seek to unfold the specific cognitive mechanisms that each brain region contributes to during learning (Zeithamova et al., 2019).

This modern approach is particularly vital because building knowledge often involves more than just extracting patterns; it also requires organizing information according to rules (Nosofsky, 1986; Ashby et al., 1998). For successful learning, one needs to discover the set of sufficient and necessary conditions such that they can map the relevant features of an item to category membership (Nosofsky, 1986; Ashby et al., 1998). However, most concepts in life are not structured based on deterministic rules and may include atypical members (Nosofsky et al., 1994; Palmeri and Nosofsky, 1995; Nosofsky and Palmeri, 1998). The ability to learn exceptions that do not follow the simple rules of a concept is what makes our knowledge flexible (Nosofsky et al., 1994; Palmeri and Nosofsky, 1995; Nosofsky and Palmeri, 1998). Computational models of concept learning such as SUSTAIN (i.e., Supervised and Unsupervised STratified Adaptive Incremental Network) suggest that exceptions require specialized representations that distinguish them from regular concept members (Love et al., 2004; Love and Gureckis, 2007). Furthermore, model-driven approaches have revealed medial temporal lobe engagement during concept formation, specifically in representing exceptions to concept rules (Davis et al., 2012a,b). This raises the intriguing question of whether the brain networks supporting flexible knowledge formation, particularly the learning of exceptions, are distinct from those used for learning rule-abiding items.

Communication across widespread brain regions is supported by white matter connections. Although their role in learning and memory has not been extensively studied, individual variability in these structures has been linked to cognitive abilities. For instance, the uncinate fasciculus, which connects the prefrontal and temporal cortices, is linked to associative learning, supporting the retrieval of accurate episodic memory representations from among the competing ones (Alm et al., 2016; Metoki et al., 2017). Resolving the conflict between competing concepts is associated with the inferior longitudinal (i.e., linking the occipital and temporal cortices) and the inferior occipitofrontal fasciculi (i.e., connecting the occipital, temporal, parietal regions) (Nugiel et al., 2016). During development, white matter structures that connect to the hippocampus, including the fornix, have been shown to support episodic memory (Ngo et al., 2017; Hoffman et al., 2022). Moreover, recent advancements in diffusion imaging have revealed that the white matter connections within the hippocampus are also related to various cognitive abilities, including concept learning (Schlichting et al., 2021; Gumus et al., 2025; Gumus and Mack, 2026). For instance, our prior work discovered that learning regularities and exceptions was supported by distinct hippocampal pathways (Gumus and Mack, 2026). However, it remains largely unexplored how white matter structures spanning a wide range of cortical and subcortical regions support concept learning. Thus, the present study explored how whole-brain white matter connections contribute to flexible concept formation, which includes both rule-following items and exceptions. Although a set of specific pathways seem to be related to learning, a broader perspective that isolates white matter networks that are important for the dynamics of flexible learning is warranted. Given that concept learning recruits a distributed set of brain regions from visual and parietal cortices to prefrontal cortex and medial temporal lobe structures, a data-driven, whole-brain approach is essential to move beyond predefined tracts, allowing potentially complementary networks to emerge based on their relationship to specific learning demands. Thus, by leveraging correlational tractography, we sought to identify white matter networks associated with rule-following and exception learning, thereby clarifying the complementary structural scaffolds that underpin flexible concept formation.

## 2 Materials and methods

### 2.1 Participants

This study is based on a previously reported dataset (Schlichting et al., 2021; Gumus and Mack, 2026). Forty-three healthy participants (24 female, 19 male) with the mean age of 23.5 ranging between 18 and 34 were recruited for this study from the University of Toronto community. All participants were right-handed, fluent English speakers with normal to corrected-to-normal vision and had no history of neurological or psychological disorders. The study was approved by University of Toronto Research Ethics Board. Informed consent was obtained from each participant. They were compensated $20/h for participating. Five participants were excluded from the analyses due to issues with experiment presentation (*N* = 1), failure to follow the task instructions (*N* = 2), or technical issues with the MRI acquisition (*N* = 2).

### 2.2 Study procedure

Each subject participated in 1.5 h long MRI scan that included a category learning task. Participants performed a category learning task in the MRI scanner located at the Toronto Neuroimaging Facility (ToNI) at the University of Toronto. Participants were able to see a screen through a mirror attached to the head coil and responded with a remote control that was handed to them by MRI technician or the study lead (M.G). Foam pads were used to minimize head motion to reduce noise in the captured neuroimages. Stimulus presentation and timing were performed using custom scripts written in Matlab (Mathworks) and Psychtoolbox.^1^

### 2.3 Category learning task

Participants performed a category learning task. In each trial, they were presented with a cartoon flower and instructed to decide whether it preferred sun or shade for better growth (Figure 1a). This task required participants to consider the features of the flowers for categorization. Specifically, the two flower categories (i.e., sun and shade) were distinguished along three feature dimensions: outer petal shape, inner petal shape and color of the flower center. Categories were defined based on a rule-plus-exception category structure (Shepard et al., 1961). Each category included an anchor (i.e., prototypical members), rule-following items (i.e., that closely followed the rules as they are 1 feature away from anchors), and an exception (i.e., violated the rules) (Figure 1a). Learning to categorize exceptions were relatively difficult in comparison to anchors and rule-following items because they were only one feature away from the anchors of the opposite category. Exceptions looked much like the anchors of the opposite category than the anchors of the category they belonged to, making them an index for flexible learning.

**FIGURE 1:**
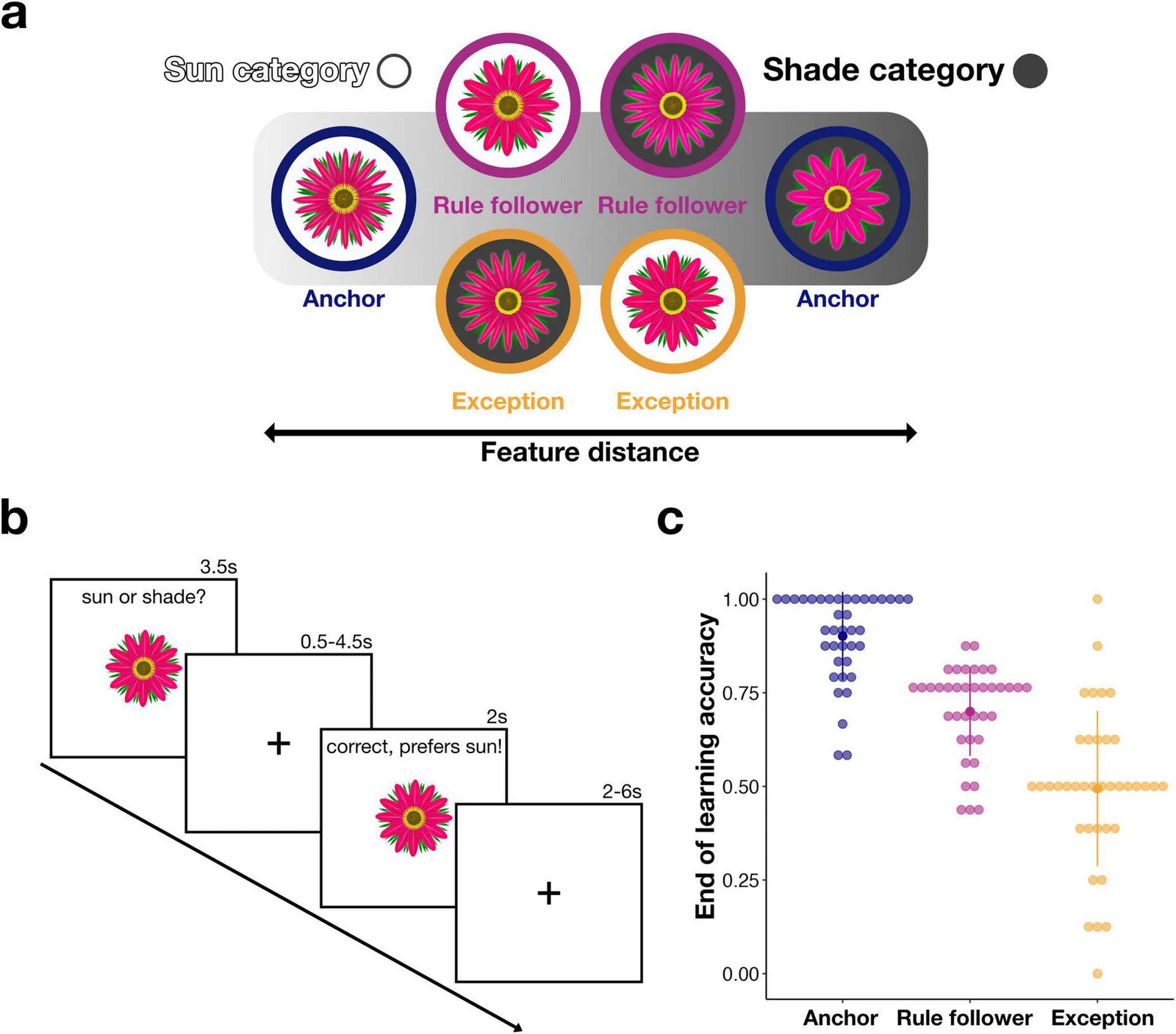
Experimental design and participant learning performances. **(a)** Flower stimuli varied in width of outer (pink) petals, shape of inner (yellow) petals, and color/texture of the brown center. Anchors were more prototypical members of the categories. Rule-following items were one feature away from anchors and closely followed the rules. Exceptions were two features away from the anchors and violated the rules. **(b)** In each trial, participants decided whether the presented flower stimulus preferred sun or shade and received feedback immediately. **(c)** Participants learned anchors very well while there was more variability in rule-followers and exceptions. Adapted from Schlichting et al. (2021), Gumus and Mack (2026).

In each trial, flower stimulus was shown for 3.5 s (Figure 1b). During this time, participants were instructed to choose if a shown flower preferred a sun or shade by pressing the remote control within the MRI scanner. The stimulus presentation was followed by a fixation cross for 0.5–4.5 s. Then, feedback screen appeared for 2 s, showing the flower stimulus again and indicating whether the response was correct or incorrect. If the response was incorrect, then the feedback also included the correct category label on the screen.

### 2.4 MRI acquisition

Whole-brain imaging data were acquired on a 3.0T Siemens Prisma system at the Toronto Neuroimaging Facility located at the University of Toronto. A high-resolution T1-weighted MPRAGE structural volume (TR = 2 s, TE = 2.4 ms, flip angle = 9°, FOV = 256 mm, matrix = 256 × 256, 1 mm isotropic voxels) was acquired for co-registration and parcellation. DWI were collected in two separate scans per participant within the same session (axial echo-planar imaging, GRAPPA = 2, multiband factor = 2, b-value = 1,000 s/mm^2^, TR = 4,000 ms, TE = 70 ms, 64 directions per run, 22 cm FOV, 2 mm-isotropic voxels, 70 slices, acquisition time = 298 s; sequence also included four repetitions of b-value = 0 images).

### 2.5 Diffusion weighted image processing

The diffusion MRI scans of 38 participants were included in the connectometry database. A High Angular Resolution Diffusion Imaging (HARDI) acquisition scheme was used. The two runs were concatenated prior to preprocessing, yielding a total of 128 diffusion-weighted directions per participant, consistent with a HARDI scheme. FSL eddy was used to correct for eddy current and motion distortions. The correction was conducted through the integrated interface in DSI Studio (“Chen” release)^2^. The accuracy of b-table orientation was examined by comparing fiber orientations with those of a population-averaged template (Yeh et al., 2018). The restricted diffusion imaging was utilized (Yeh et al., 2017). The diffusion data were reconstructed using generalized q-sampling imaging (GQI; Yeh et al., 2010) with a diffusion sampling length ratio of 1.25. The tensor metrics were calculated from the b-value = 1,000 volumes. Diffusion data were reconstructed using GQI to better resolve complex fiber configurations. Fractional anisotropy (FA) values were extracted from the tensor model and used as the tract-based metric in the connectometry analysis. FA quantifies the degree to which water diffusion is directionally constrained within a voxel, serving as an index of white matter microstructural integrity.

### 2.6 Connectometry analyses

Diffusion MRI connectometry (Yeh et al., 2016) was used to isolate whole-brain tractography that correlated with individual differences in learning performance. Specifically, FA in white matter structures throughout the brain was correlated with end-of-learning accuracy separately for category rule followers and exceptions. The correlational tractography was based on a nonparametric Spearman partial correlation with effects of sex and age included as potentially confounding variables. To identify white matter networks specifically associated with exception learning, rule-following performance was included as a control variable in the partial correlation analysis; and vice versa for the separate analysis to identify rule-following learning.

Standard, widely adopted parameters were used for the connectometry analyses conducted in DSI Studio (Yeh et al., 2016). A T-score threshold of 2.5 was assigned and tracked using a deterministic fiber tracking algorithm (Yeh et al., 2013) to obtain correlational tractography. Critically, this threshold serves as a tract-building criterion such that only tracking points along streamlines exceeding T ≥ 2.5 contribute to tract formation rather than a traditional significance threshold. Statistical validity is then evaluated based on the length of identified tracts against an empirical null distribution of tract lengths generated by permutation, such that surviving tracts must reflect spatially contiguous structure-behavior relationships along their length. Whole brain seeding was used. The tracts were filtered by topology-informed pruning (Yeh et al., 2019) with 16 iterations. An FDR threshold of 0.05 was used to select tracts. To estimate the false discovery rate, a total of 4,000 randomized permutations were applied to randomize the variable of interest to obtain the null distribution of the tract length. For visualization of the relationship between resulting networks and learning ability, we estimated two separate linear models depicting how each stimulus type (i.e., anchors, rule-following items, and exceptions) relates to FA of the resulting networks. This additional analysis was post-hoc and intended solely for visualization, rather than for statistical inference.

## 3 Results

### 3.1 Learning performance

Participants learned anchors very well (the end of learning accuracy: *M* = 89.49%, SD = ±13.10) while rule-follower performance was lower (*M* = 68.17%, SD = ±12.84) and exception learning was the lowest but also highly variable (*M* = 47.52%, SD = ±16.65) (Figure 1c). This learning behavior was previously reported (Schlichting et al., 2021; Gumus and Mack, 2026). The variability observed in rule-follower and exception learning encouraged us to examine how different learning demands (i.e., rule-following items versus exceptions) might be linked to distinct white matter networks.

### 3.2. Distinct white matter networks emerge for learning rule followers and exceptions

We discovered that learning the items that closely followed the rules relied on a distinct white matter network than that of exceptions (Figure 2). While exception network included various sub-cortical tracts (Supplementary Table 1), rule-following network included several long-range white matter tracts (Supplementary Table 2). Three main tract bundles – namely corpus collosum body, corpus collosum tapetum, and right superior longitudinal fasciculus – were observed in both exception and rule-following networks and thus were labeled as overlapping network (Figure 2 and Supplementary Table 3).

**FIGURE 2:**
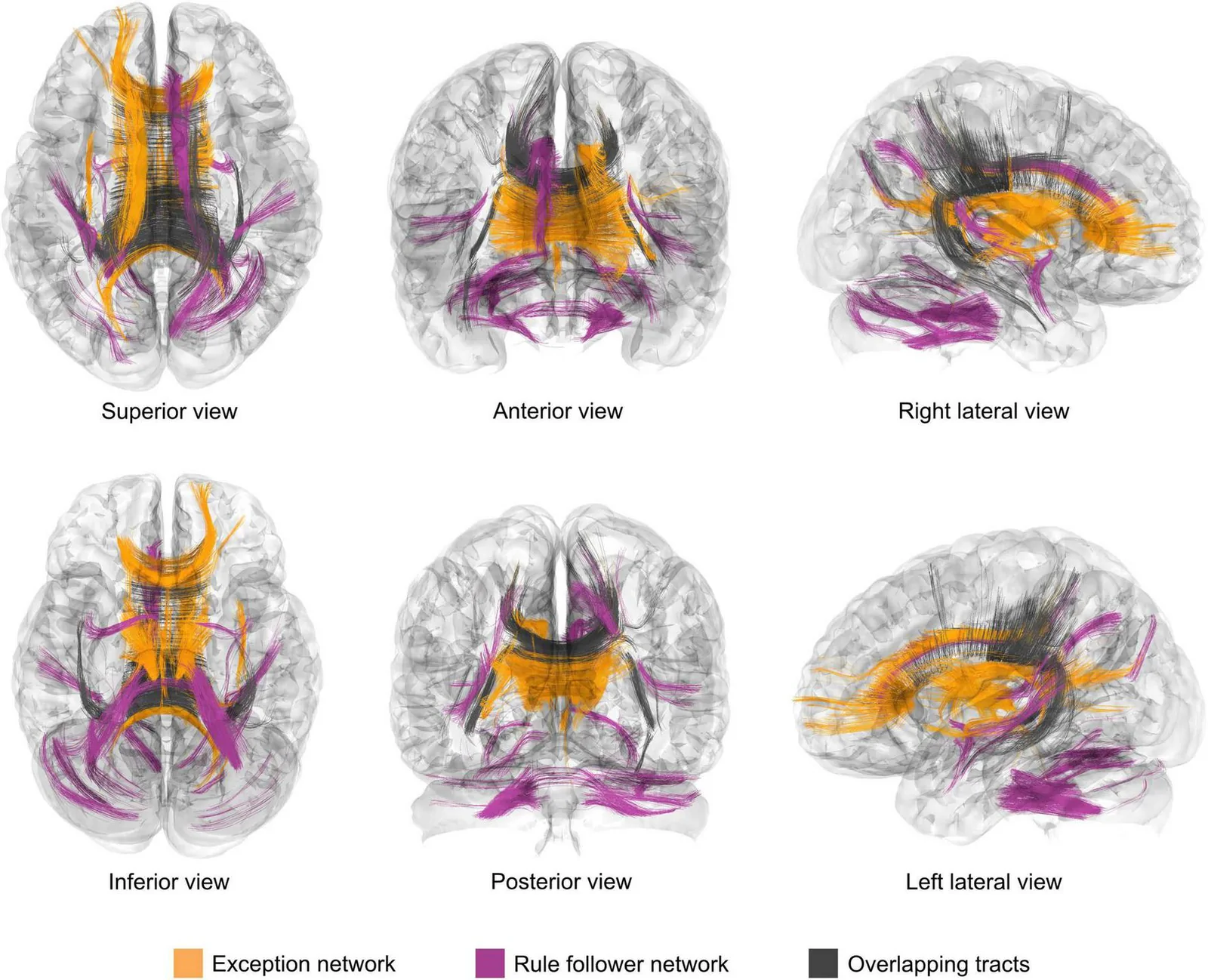
Complementary white matter networks in learning rule-followers and exceptions. Whole-brain correlational tractography analyses resulted in two distinct white matter networks where fractional anisotropy of the tract bundles in a network was significantly correlated with learning accuracy. The white matter bundles associated with learning rule-following items is shown in purple while white matter bundles related to exception learning was depicted in orange. Tract bundles that appeared in both rule-following and exception networks are demonstrated in black. The multiple views illustrate the distinct anatomical locations of these distinct networks.

#### 3.3 Exception network

A distinct network of tracts was uniquely associated with the successful learning of category exceptions (FDR ≤ 0.05) that emerged from the correlational tractography analyses. Higher accuracy in identifying exception items was significantly correlated with greater FA in multiple tracts including the bilateral fornix, bilateral superior and anterior thalamic radiations, forceps minor, and the frontal-parietal portion of the left cingulum (Figure 3a). Full list of tract bundles in exception network is listed in Supplementary Table 1. As shown in Figure 3b, there was a significant positive correlation between the mean FA within this exception network and learning accuracy for exception items (β = 0.12, 95% CI [0.07, 0.17], *p* < 0.001), which was computed only for visualization purposes. The mean FA of this network was not corelated with learning rule-followers (β = 0.07, 95% CI [−0.02, 0.15], *p* = 0.12) or anchors (β = −0.009, 95% CI [−0.09, 0.07], *p* = 0.83), emphasizing its unique relation to exception learning. These findings suggest that distinct structural pathways underpin the cognitive processes required for learning predictable patterns versus handling novel, atypical information.

**FIGURE 3:**
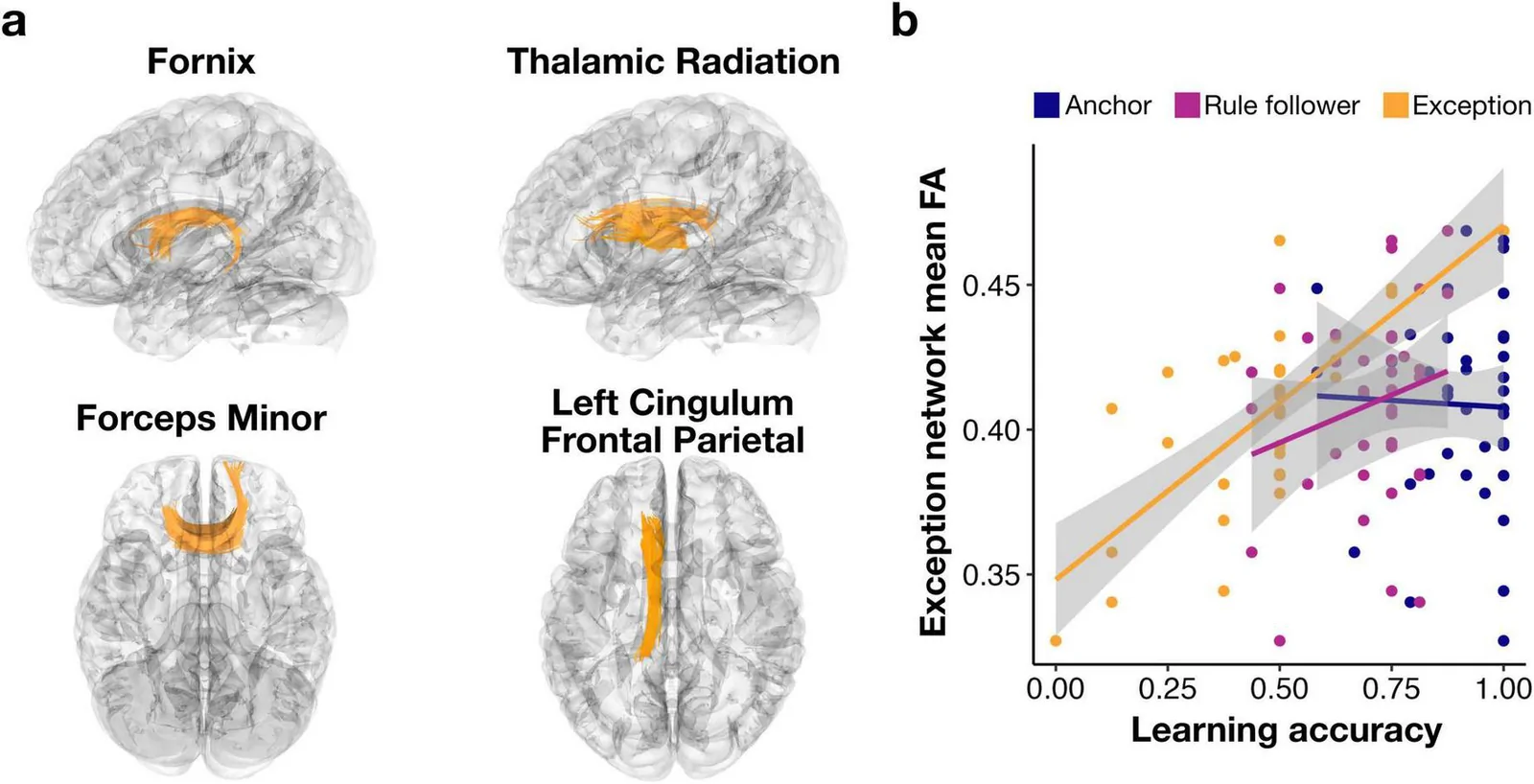
The exception network spans subcortical regions including thalamus, hippocampus, but also frontal lobe. **(a)** A sample of white matter bundles that were uniquely associated with exception learning were depicted. The exception network included several tract bundles such as the fornix, thalamic radiations, forceps minor, and the frontal-parietal portion of the left cingulum bundle. **(b)** The significant correlation between the mean FA of this network and exception learning is depicted along with learning performance for rule-followers and anchors. Each dot represents a participant for a specific item type learning and the mean FA of the exception network.

### 3.4 Rule follower network

The correlational tractography analyses yielded a significant positive relationship between learning accuracy for rule-following items and the integrity of a distributed network of white matter tracts (FDR ≤ 0.05). Specifically, higher performance on these items was associated with greater FA in multiple tracts including the right frontal-parietal cingulum, right parahippocampal parietal cingulum, bilateral middle longitudinal fasciculus, and middle cerebellar peduncle (Figure 4a). Full list of tracts bundles in rule-following network is listed in Supplementary Table 2. The strength of this relationship is illustrated in Figure 4b, which shows a positive correlation between mean FA across this network and learning accuracy for rule-following items (β = 0.14, 95% CI [0.07, 0.22], *p* < 0.001). The mean FA of this network was not corelated with leaning exceptions (β = 0.03, 95% CI [−0.01, 0.07], *p* = 0.18) or anchors (β = −0.04, 95% CI [−0.03, 0.12], *p* = 0.25), emphasizing its unique relation to rule-following items.

**FIGURE 4:**
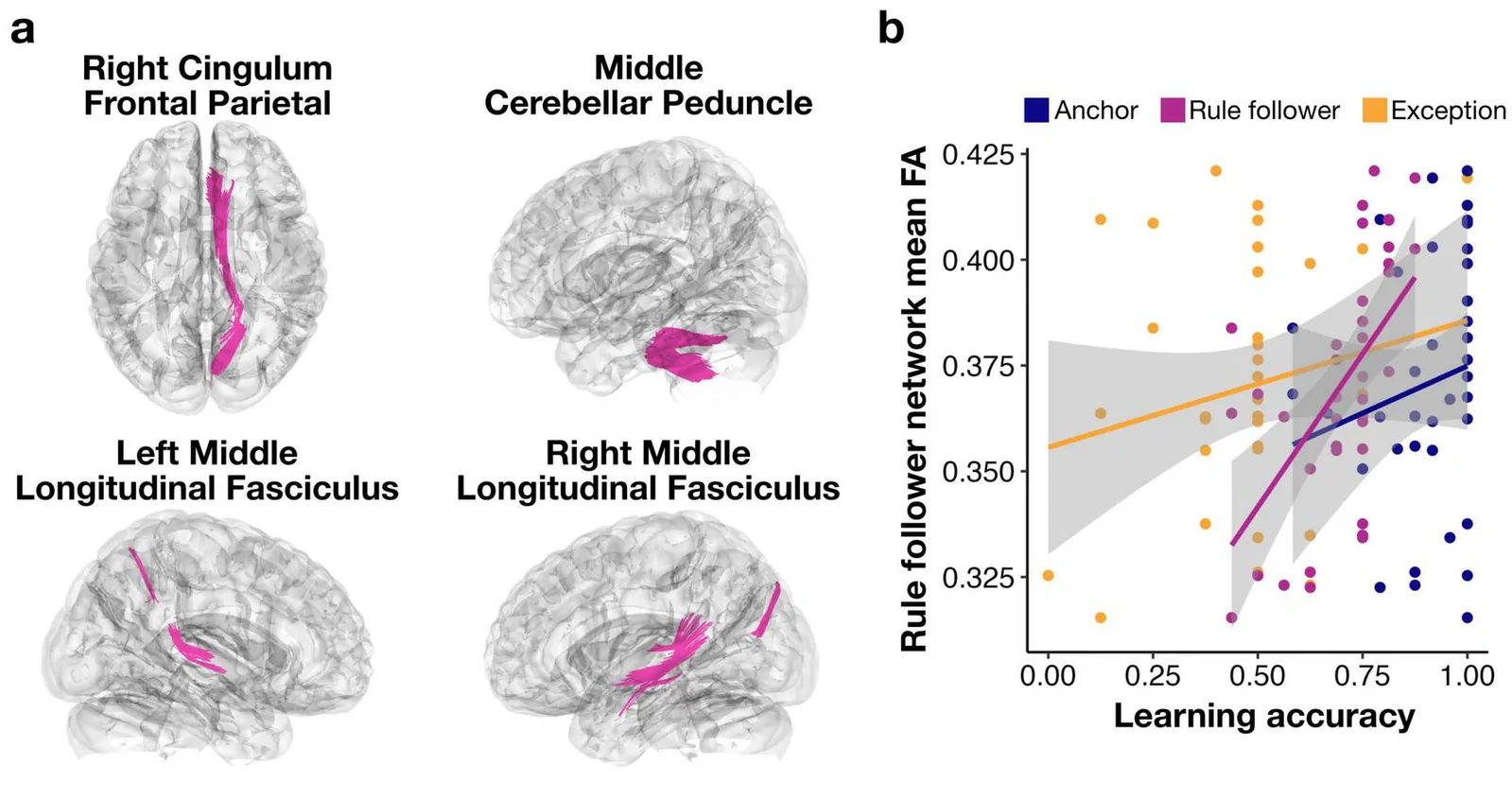
The rule-follower network consists of long-range white matter and cerebellar tracts. **(a)** A sample of white matter bundles that were uniquely associated with learning performance for rule-following items were depicted. The rule-following network included several long-range white matter bundles such as the frontal-parietal portion of the right cingulum bundle, the bilateral middle longitudinal fasciculus, and the middle cerebellar peduncle. **(b)** The significant correlation between the mean FA of this white matter network and learning performance for rule-following items was plotted along with learning performance for exceptions and anchors. Each dot represents a participant with their learning performance for a specific stimulus type and the mean FA of the rule-follower network.

## 4 Discussion

Flexible concept formation relies on balancing two competing cognitive demands: abstracting generalizable patterns (i.e., rules) while simultaneously retaining specific instances that violate them (i.e., exceptions). Converging neural and computational evidence suggests that these two processes are functionally dissociable, with distinct brain regions and mechanisms contributing to each (Love et al., 2004; Davis et al., 2012a; Mack et al., 2016). Extending this work, diffusion imaging has further revealed that these complementary learning demands are underpinned by distinct hippocampal white matter pathways (Schlichting et al., 2021; Gumus and Mack, 2026). Contrasting these two learning demands therefore provides a theoretically guided framework for investigating the broader white matter networks that underlie flexible concept formation. We leveraged whole-brain correlational tractography to investigate the structural architecture that underlies this dual capacity in concept learning. Our findings provide novel evidence that learning these distinct item types, namely rule-followers versus exceptions, is supported by anatomically dissociable white matter networks. Specifically, learning items that conformed to category rules was associated with the integrity of long-range cortical association tracts, including the right frontal-parietal cingulum, bilateral middle longitudinal fasciculus, and the middle cerebellar peduncle. In contrast, learning exceptions relied on a predominantly subcortical-limbic network, involving connections such as the fornix and thalamic radiations, alongside cortical tracts such as the forceps minor and the left frontal-parietal cingulum. These results underscore the idea that the flexible concept formation is supported by dissociable white matter anatomies, each uniquely suited for distinct computational demands.

The white matter network associated with successful exception learning points toward a system specialized for encoding unique, detailed information. The involvement of the fornix, the primary hippocampal output tract, is highly consistent with prior research implicating the hippocampus in learning exceptions (Davis et al., 2012a; Schlichting et al., 2021) and encoding novel or specific episodic details (Mack et al., 2016, 2018). Crucially, individual variability in fornix microstructure has previously been linked to the ability to discriminate between similar episodic events (Postans et al., 2014; Bennett et al., 2015), a process analogous to resolving the interference between rule-followers and exceptions. Specifically, the hippocampus is thought to use pattern separation to create distinct memory traces for unique exemplars (Yassa and Stark, 2011); our results suggest that the integrity of the fornix might be critical for transmitting these pattern-separated representations to the cortex. Indeed, while our previous work linked intrinsic hippocampal white matter integrity to exception learning (Schlichting et al., 2021; Gumus and Mack, 2026), the current finding extends this by highlighting a critical connection between the hippocampus and the rest of the brain. Specifically, the role of the hippocampus in learning exceptions, in other words handling atypical exemplars and their separation from typical items (Hutter and Wilson, 2018), might be relying on a robust output via the fornix to broadcast prediction errors to the wider cortical network.

As part of the exception network, the thalamic radiations likely reflect the structural scaffold of the extended hippocampal system (Aggleton and Brown, 1999; Aggleton and O’Mara, 2022), specifically linking the anterior thalamus, also a major target of the fornix, to the prefrontal cortex. Recent diffusion imaging indicates that the integrity of thalamic radiations relates to episodic memory fidelity (De Simone et al., 2024), hinting at its role as a conduit for relaying exemplar-specific information from the hippocampus to executive regions. Since exceptions require overcoming prepotent rule-based responses to focus on distinguishing features, this pathway becomes crucial. While the thalamus generally filters sensory inputs (Leszczyński and Staudigl, 2016), the hippocampal-thalamic-prefrontal connection may specifically distribute the neural signal of a surprising, exceptional item, prioritizing it for higher-level processing. To handle these distinct items, the brain must inhibit dominant rules and actively maintain atypical representations (Freedman et al., 2002, 2003). Here, the forceps minor likely plays a central role; by connecting bilateral prefrontal cortices, it facilitates the interhemispheric communication necessary to resolve the conflict between rule and exemplar (Miller et al., 2002). Supporting this process is the left cingulum bundle (frontal-parietal portion), which offers a structural basis for error monitoring. As the primary connection to the left anterior cingulate cortex, a region critical for detecting conflict (Swick and Turken, 2002), the cingulum’s integrity might facilitate recognizing the mismatch between an expected rule-based outcome and the actual exception. Collectively, these findings suggest that the exception network is well-suited to support the detailed mnemonic encoding and cognitive control demanded by atypical exemplars.

The network associated with learning rule-following items suggests a system optimized for establishing and maintaining generalizable knowledge. The right cingulum bundle (frontal-parietal portion) likely supports active retention and consistent application of the category rule across trials. This interpretation aligns with models of cognitive control where frontal-parietal connections are essential for actively sustaining “task sets”; the specific stimulus-response mappings required for rule-based behavior (Miller and Cohen, 2001). Working in concert with this control mechanism, the bilateral middle longitudinal fasciculus likely provides the structural scaffold for abstraction. By connecting parietal and occipital regions to the anterior temporal lobe, its integrity would be crucial for integrating visual features (occipital; Folstein et al., 2013) and their spatial configurations (parietal; Fitzgerald et al., 2012) with stored conceptual knowledge and rule representations (anterior temporal lobe; Hoffman and Lambon Ralph, 2018; Martin, 2007). This structural integration might underpin the cognitive process of abstraction (Ralph et al., 2017), allowing the system to look past the features of a single item and extract the commonalities that define a category rule. Consequently, the integrity of these long-range tracts within the rule-follower network thus might support the global integration of features, enabling the efficient, automated classification of typical rule-abiding items.

The involvement of the middle cerebellar peduncle, the principal afferent pathway to the cerebellum, in the rule-follower network, is intriguing but warrants nuanced interpretation. The cerebellum is traditionally associated with motor control, and thus some portion of this association may reflect the manual response demands of our categorisation task. However, converging evidence implicates the cerebellum in higher cognitive operations, and clinical studies demonstrate that cerebellar disruption impairs non-motor implicit learning and cognitive associative processing (Bellebaum and Daum, 2007; Baillieux et al., 2008; Timmann et al., 2010). In the present context, the microstructural integrity of this tract may index the efficiency of cortico-cerebellar communication supporting the gradual proceduralization of rule-based categorization across trials, which is a mechanism aligned with theoretical frameworks proposing that cerebellar circuits help automate routine mental operations (Ramnani, 2006; Balsters and Ramnani, 2011; Stoodley, 2012; Koziol et al., 2014). However, we note that direct evidence linking cerebellar tracts specifically to category rule learning remains limited. Future work combining tractography with functional connectivity will be essential to disentangle these potential cognitive contributions of cerebellar tracts from general motor execution demands.

Beyond the learning-specific networks, three white matter tracts were shared across both conditions: the corpus callosum body, the corpus callosum tapetum, and the right superior longitudinal fasciculus. While the specific contributions of these tracts to concept learning remain to be fully mapped, their joint involvement likely reflects baseline attentional and perceptual demands common to both item types. The right superior longitudinal fasciculus connects frontal, parietal, and temporal regions and is central to top-down visuospatial attention (de Schotten et al., 2011), likely indicates a capacity engaged regardless of item category. Similarly, the corpus callosum body facilitates interhemispheric coordination during cognitively demanding tasks (Schulte and Müller-Oehring, 2010). Lastly, the corpus callosum tapetum connects bilateral temporal regions, forming the lateral extension of posterior splenial fibers into temporal and occipital cortices (Wakana et al., 2004). While functional literature conventionally attributes visual interhemispheric transfer to the splenium as a whole (Berlucchi, 2014), the tapetum provides the direct structural link between temporal lobes required for bilateral feature integration. It may thus serve as the structural pathway for exchanging complex feature information between temporal lobes, though functional neuroimaging studies rarely isolate the tapetum from the main body of the splenium. Together, these shared tracts may provide a domain-general structural scaffold for basic attentional and visual integration, upon which specialized learning networks operate. This interpretation is broadly consistent with recent functional white matter parcellations (Zhou et al., 2026), which demonstrates that posterior callosal subregions, including the isthmus and splenium, anatomically proximate to the tapetum, exhibit functional connectivity with frontoparietal, visual, and default mode networks, while the callosal midbody connects predominantly with cerebellar and brainstem networks, reflecting the broad visuospatial and coordinative demands shared across both learning conditions.

The dissociation between these two networks provides a structural basis for the flexible nature of concept learning. One system, reliant on hippocampal outputs and prefrontal control, appears primed for encoding novel, unique, or conflicting information such as the exceptions. The other, leveraging long-range cortico-cortical and cortico-cerebellar pathways, seems to support the extraction of regularities and the efficient application of established rules. This architectural division aligns with multiple-systems theories of category learning, which propose that a rule-based system and an exemplar-based system operate in parallel (Ashby et al., 1998; Nomura and Reber, 2008). While previous research has focused heavily on the functional dissociation of these systems, our results demonstrate that this division of labor is deeply rooted in the structural connectivity. This structural separation likely allows the cognitive system to be both efficient in processing familiar patterns and robust in adapting to new, atypical information (Zeithamova et al., 2019). Furthermore, these findings suggest that individual differences in learning strategies are not merely functional choices but are underpinned by structural connectivity (Scholz et al., 2009). Just as white matter plasticity has been observed in learning (Hofstetter et al., 2013; Sampaio-Baptista et al., 2013), the structural variation observed here likely underlies the learning strategy with which individuals acquire new concepts, specifically deploying rule abstraction versus encoding of specific instances.

This study provides evidence for the involvement of complementary white matter networks in flexible concept learning, but several limitations exist. While our correlational tractography successfully identified distinct white matter networks (one cortico-cortical for rule learning, one predominantly subcortical for exception learning), these findings only establish the necessary anatomical substrate for flexible concept formation. We had to infer the functional role of white matter bundles within these networks based on existing literature. Specifically, the interpretations we propose, wherein the subcortical network mediates experience-based processing (exceptions), and the cortical network supports explicit feature integration (rules), is speculative with respect to the functional dynamics and thus should be further explored. One limitation of the present study is that independent neuropsychological batteries measuring domain-general cognitive capacities such as working memory capacity, attentional control, and executive function were not collected. While our paradigm directly indexed task-specific rule and exception learning performance, future research incorporating standardized cognitive assessments will be valuable to determine how baseline executive capacities interact with white matter microstructural integrity to modulate flexible concept formation. A separate methodological limitation relates to our exploratory tract-specific analysis approach. Correlational tractography methods, while powerful for discovery and minimizing *a priori* bias, define the relevant white matter bundles based purely on their statistical relationship with behavior. This data-driven method can lead to tracts that are partial segments of larger, well-established bundles, making their relationship to classical neuroanatomy difficult. For instance, the networks identified may represent only the most robust segments of broader pathways, potentially excluding adjacent fibers that also contribute to the network, or conflating segments of distinct, neighboring bundles. Future research should prioritize a hypothesis-driven approach, such as fiber tracking with specific anatomical region-of-interest, informed by our current findings. This targeted approach would allow for the precise anatomical isolation and characterization of the underlying structural networks, providing a clearer explanation of how well-defined white matter architecture supports the proposed rule and exception processing systems. Considerations regarding sample size should also be noted. Although *N* = 38 is consistent with published connectometry literature employing similar DSI Studio frameworks (Sorg et al., 2021; Hodgdon et al., 2022; Park et al., 2024), modest sample sizes warrant careful interpretation. Statistical validity is anchored by the 4,000-iteration permutation procedure and tract-length false discovery rate correction (q < 0.05), which empirically controls for false positives along contiguous white matter pathways. *Post hoc* influence analyses further confirmed that results were robust to the removal of influential participants, with effect sizes remaining significant and comparable in magnitude after their exclusion. Nevertheless, future studies with larger independent cohorts will be valuable to confirm the population-level generalizability of these findings. Furthermore, future work would benefit from formally situating connectometry findings within emerging multimodal frameworks that integrate functional and diffusion metrics to characterize white matter network affiliations (Zhou et al., 2026). Precedent for combining these modalities exists within the domain of concept learning as our prior work demonstrated that integrating diffusion-based pathway estimates with task-based functional activation reveals dynamic learning-related engagement of white matter pathways that neither modality captures alone (Gumus and Mack, 2026). Extending this multimodal approach to whole-brain white matter networks represents a promising direction for disambiguating domain-general from learning-specific structural contributions.

In conclusion, our findings provide novel evidence that flexible concept formation is underpinned by a structural dissociation within white matter architecture. We identified two complementary networks: a predominantly subcortical (i.e., including the fornix, thalamic radiations, forceps minor, and left cingulum) supporting the processing of exceptions, and a cortico-cortical network (i.e., featuring the right cingulum, middle longitudinal fasciculus, and middle cerebellar peduncle) supporting the learning of items abiding category rules. This structural organization suggests a neuroanatomical basis for the dynamic functional interplay between established cortical systems and memory-driven subcortical systems required for cognitive flexibility. Ultimately, these results highlight the critical role of specific white matter pathways in supporting the complex, dual-process cognition that allows us to build and adapt our conceptual understanding of the world, providing a foundational neuroanatomical blueprint for understanding individual variability in flexible concept learning.

## Data Availability

The datasets presented in this study can be found in online repositories. The names of the repository/repositories and accession number(s) can be found below: please see our previously published work (Gumus and Mack, 2026; Schlichting et al., 2021).

## Footnotes

1
www.psychtoolbox.org

2
http://dsi-studio.labsolver.org

## References

[B1] Aggleton J. P. Brown M. W. (1999). Episodic memory, amnesia, and the hippocampal-anterior thalamic axis. *Behav. Brain Sci.* 22, 425–444. 10.1017/S0140525X99002034 11301518

[B2] Aggleton J. P. O’Mara S. M. (2022). The anterior thalamic nuclei: Core components of a tripartite episodic memory system. *Nat. Rev. Neurosci*. 23, 505–516. 10.1038/s41583-022-00591-8 35478245

[B3] Alm K. H. Rolheiser T. Olson I. R. (2016). Inter-individual variation in fronto-temporal connectivity predicts the ability to learn different types of associations. *Neuroimage* 132, 213–224. 10.1016/j.neuroimage.2016.02.038 26908315 PMC4851884

[B4] Aly M. Turk-Browne N. B. (2016). Attention promotes episodic encoding by stabilizing hippocampal representations. *Proc. Natl. Acad. Sci. U. S. A*. 113, E420–E429. 10.1073/pnas.1518931113 PMC4743819 26755611

[B5] Ashby F. G. Alfonso-Reese L. A. Turken A. U. Waldron E. M. (1998). A neuropsychological theory of multiple systems in category learning. *Psychol. Rev*. 105, 442–481. 10.1037/0033-295x.105.3.442 9697427

[B6] Ashby F. G. Maddox W. T. (2005). Human category learning. *Annu. Rev. Psychol*. 56, 149–178. 10.1146/annurev.psych.56.091103.070217 15709932

[B7] Ashby F. G. Maddox W. T. (2011). Human category learning 2.0. *Ann. N. Y. Acad. Sci*. 1224, 147–161. 10.1111/j.1749-6632.2010.05874.x 21182535 PMC3076539

[B8] Baillieux H. De Smet H. J. Paquier P. F. De Deyn P. P. Mariën P. (2008). Cerebellar neurocognition: Insights into the bottom of the brain. *Clin. Neurol. Neurosurg*. 110, 763–773. 10.1016/j.clineuro.2008.05.013 18602745

[B9] Balsters J. H. Ramnani N. (2011). Cerebellar plasticity and the automation of first-order rules. *J. Neurosci*. 31, 2305–2312. 10.1523/JNEUROSCI.4358-10.2011 21307266 PMC6633030

[B10] Bellebaum C. Daum I. (2007). Cerebellar involvement in executive control. *Cerebellum* 6, 184–192. 10.1080/14734220601169707 17786814

[B11] Bennett I. J. Huffman D. J. Stark C. E. (2015). Limbic tract integrity contributes to pattern separation performance across the lifespan. *Cereb. Cortex*. 25, 2988–2999. 10.1093/cercor/bhu093 24825784 PMC4553719

[B12] Berlucchi G. (2014). Visual interhemispheric communication and callosal connections of the occipital lobes. *Cortex* 56, 1–13. 10.1016/j.cortex.2013.02.001 23489777

[B13] Bowman C. R. Zeithamova D. (2018). Abstract memory representations in the ventromedial prefrontal cortex and hippocampus support concept generalization. *J. Neurosci*. 38, 2605–2614. 10.1523/JNEUROSCI.2811-17.2018 29437891 PMC5858598

[B14] Constantinescu A. O. O’Reilly J. X. Behrens T. E. J. (2016). Organizing conceptual knowledge in humans with a gridlike code. *Science* 352, 1464–1468. 10.1126/science.aaf0941 27313047 PMC5248972

[B15] Corbetta M. Shulman G. L. (2002). Control of goal-directed and stimulus-driven attention in the brain. *Nat. Rev. Neurosci*. 3, 201–215. 10.1038/nrn755 11994752

[B16] Corbetta M. Miezin F. M. Dobmeyer S. Shulman G. L. Petersen S. E. (1991). Selective and divided attention during visual discriminations of shape, color, and speed: Functional anatomy by positron emission tomography. *J. Neurosci*. 11, 2383–2402. 10.1523/JNEUROSCI.11-08-02383.1991 1869921 PMC6575512

[B17] Davis T. Love B. C. Preston A. R. (2012a). Learning the exception to the rule: Model-based FMRI reveals specialized representations for surprising category members. *Cereb. Cortex*. 22, 260–273. 10.1093/cercor/bhr036 21666132

[B18] Davis T. Love B. C. Preston A. R. (2012b). Striatal and hippocampal entropy and recognition signals in category learning: Simultaneous processes revealed by model-based fMRI. *J. Exp. Psychol. Learn. Mem. Cogn*. 38, 821–839. 10.1037/a0027865 22746951 PMC3403290

[B19] de Schotten M. T. Dell’Acqua F. Forkel S. J. Simmons A. Vergani F. Murphy D. G. et al. (2011). A lateralized brain network for visuospatial attention. *Nat. Neurosci*. 14, 1245–1246. 10.1038/nn.2905 21926985

[B20] De Simone M. S. Spalletta G. Vecchio D. Bassi A. Carlesimo G. A. Piras F. (2024). The role of the anterior thalamic nuclei in the genesis of memory disorders in Alzheimer’s disease: An exploratory study. *J. Alzheimers Dis*. 97, 507–519. 10.3233/JAD-230606 38189755

[B21] Fitzgerald J. K. Swaminathan S. K. Freedman D. J. (2012). Visual categorization and the parietal cortex. *Front. Integr. Neurosci*. 6:18. 10.3389/fnint.2012.00018 22582040 PMC3348721

[B22] Foerde K. Knowlton B. J. Poldrack R. A. (2006). Modulation of competing memory systems by distraction. *Proc. Natl. Acad. Sci. U. S. A*. 103, 11778–11783. 10.1073/pnas.0602659103 16868087 PMC1544246

[B23] Folstein J. R. Palmeri T. J. Gauthier I. (2013). Category learning increases discriminability of relevant object dimensions in visual cortex. *Cereb. Cortex*. 23, 814–823. 10.1093/cercor/bhs067 22490547 PMC3593573

[B24] Frank L. E. Bowman C. R. Zeithamova D. (2019). Differential functional connectivity along the long axis of the hippocampus aligns with differential role in memory specificity and generalization. *J. Cogn. Neurosci*. 31, 1958–1975. 10.1162/jocn_a_01457 31397613 PMC8080992

[B25] Freedman D. J. Assad J. A. (2006). Experience-dependent representation of visual categories in parietal cortex. *Nature* 443, 85–88. 10.1038/nature05078 16936716

[B26] Freedman D. J. Riesenhuber M. Poggio T. Miller E. K. (2002). Visual categorization and the primate prefrontal cortex: Neurophysiology and behavior. *J. Neurophysiol*. 88, 929–941. 10.1152/jn.2002.88.2.929 12163542

[B27] Freedman D. J. Riesenhuber M. Poggio T. Miller E. K. (2003). A comparison of primate prefrontal and inferior temporal cortices during visual categorization. *J. Neurosci*. 23, 5235–5246. 10.1523/JNEUROSCI.23-12-05235.2003 12832548 PMC6741148

[B28] Gilboa A. Marlatte H. (2017). Neurobiology of schemas and schema-mediated memory. *Trends Cogn. Sci*. 21, 618–631. 10.1016/j.tics.2017.04.013 28551107

[B29] Gumus M. Mack M. L. (2026). Distinct contributions of hippocampal pathways in learning regularities and exceptions revealed by functional footprints. *Proc. Natl. Acad. Sci. U. S. A*. 123:e2503388123. 10.1073/pnas.2503388123 41543896 PMC12818569

[B30] Gumus M. Bourganos A. Mack M. L. (2025). In vivo quantification of white matter pathways in the human hippocampus. *Hum. Brain Mapp*. 46:e70417. 10.1002/hbm.70417 41287444 PMC12644930

[B31] Hodgdon E. A. Courtney K. E. Yan M. Yang R. Alam T. Walker J. C. et al. (2022). White matter integrity in adolescent irritability: A preliminary study. *Psychiatry Res. Neuroimaging*. 324:111491. 10.1016/j.pscychresns.2022.111491 35635933 PMC9676048

[B32] Hoffman L. J. Ngo C. T. Canada K. L. Pasternak O. Zhang F. Riggins T. et al. (2022). The fornix supports episodic memory during childhood. *Cereb. Cortex*. 32, 5388–5403. 10.1093/cercor/bhac022 35169831 PMC9712741

[B33] Hoffman P. Lambon Ralph M. A. (2018). From percept to concept in the ventral temporal lobes: Graded hemispheric specialisation based on stimulus and task. *Cortex* 101, 107–118. 10.1016/j.cortex.2018.01.015 29475076 PMC5885984

[B34] Hofstetter S. Tavor I. Tzur Moryosef S. Assaf Y. (2013). Short-term learning induces white matter plasticity in the fornix. *J. Neurosci*. 33, 12844–12850. 10.1523/JNEUROSCI.4520-12.2013 23904619 PMC6618548

[B35] Hutter S. A. Wilson A. I. (2018). A novel role for the hippocampus in category learning. *J. Neurosci*. 38, 6803–6805. 10.1523/JNEUROSCI.1085-18.2018 30995610 PMC6596119

[B36] Koziol L. F. Budding D. Andreasen N. D’Arrigo S. Bulgheroni S. Imamizu H. et al. (2014). Consensus paper: The cerebellum’s role in movement and cognition. *Cerebellum* 13, 151–177. 10.1007/s12311-013-0511-x 23996631 PMC4089997

[B37] Kruschke J. K. (1992). ALCOVE: An exemplar-based connectionist model of category learning. *Psychol. Rev*. 99, 22–44. 10.1037/0033-295x.99.1.22 1546117

[B38] Kuhl B. A. Chun M. M. (2014). Successful remembering elicits event-specific activity patterns in lateral parietal cortex. *J Neurosci*. 34, 8051–8060. 10.1523/JNEUROSCI.4328-13.2014 24899726 PMC4044259

[B39] Kumaran D. Summerfield J. J. Hassabis D. Maguire E. A. (2009). Tracking the emergence of conceptual knowledge during human decision making. *Neuron* 63, 889–901. 10.1016/j.neuron.2009.07.030 19778516 PMC2791172

[B40] Leszczyński M. Staudigl T. (2016). Memory-guided attention in the anterior thalamus. *Neurosci. Biobehav. Rev*. 66, 163–165. 10.1016/j.neubiorev.2016.04.015 27130694

[B41] Love B. C. Gureckis T. M. (2007). Models in search of a brain. *Cogn. Affect Behav. Neurosci*. 7, 90–108. 10.3758/cabn.7.2.90 17672381

[B42] Love B. C. Medin D. L. Gureckis T. M. (2004). SUSTAIN: A network model of category learning. *Psychol. Rev*. 111, 309–332. 10.1037/0033-295X.111.2.309 15065912

[B43] Luo X. Roads B. D. Love B. C. (2021). The costs and benefits of goal-directed attention in deep convolutional neural networks. *Comput. Brain. Behav*. 4, 213–230. 10.1007/s42113-021-00098-y 34723095 PMC8550459

[B44] Mack M. L. Love B. C. Preston A. R. (2016). Dynamic updating of hippocampal object representations reflects new conceptual knowledge. *Proc. Natl. Acad. Sci. U. S. A*. 113, 13203–13208. 10.1073/pnas.1614048113 27803320 PMC5135299

[B45] Mack M. L. Love B. C. Preston A. R. (2018). Building concepts one episode at a time: The hippocampus and concept formation. *Neurosci. Lett*. 680, 31–38. 10.1016/j.neulet.2017.07.061 28801273 PMC5803467

[B46] Mack M. L. Love B. C. Preston A. R. (2026). Distinct hippocampal mechanisms support concept formation and updating. *J. Neurosci*. 46:e2077252026. 10.1523/JNEUROSCI.2077-25.2026 41781324 PMC13055146

[B47] Mack M. L. Preston A. R. Love B. C. (2020). Ventromedial prefrontal cortex compression during concept learning. *Nat. Commun*. 11:46. 10.1038/s41467-019-13930-8 31911628 PMC6946809

[B48] Malach R. Reppas J. B. Benson R. R. Kwong K. K. Jiang H. Kennedy W. A. et al. (1995). Object-related activity revealed by functional magnetic resonance imaging in human occipital cortex. *Proc. Natl. Acad. Sci. U. S. A*. 92, 8135–8139. 10.1073/pnas.92.18.8135 7667258 PMC41110

[B49] Mante V. Sussillo D. Shenoy K. V. Newsome W. T. (2013). Context-dependent computation by recurrent dynamics in prefrontal cortex. *Nature* 503, 78–84. 10.1038/nature12742 24201281 PMC4121670

[B50] Martin A. (2007). The representation of object concepts in the brain. *Annu. Rev. Psychol*. 58, 25–45. 10.1146/annurev.psych.57.102904.190143 16968210

[B51] Metoki A. Alm K. H. Wang Y. Ngo C. T. Olson I. R. (2017). Never forget a name: White matter connectivity predicts person memory. *Brain Struct. Funct*. 222, 4187–4201. 10.1007/s00429-017-1458-3 28646241 PMC5884066

[B52] Miller E. K. Cohen J. D. (2001). An integrative theory of prefrontal cortex function. *Annu. Rev. Neurosci*. 24, 167–202. 10.1146/annurev.neuro.24.1.167 11283309

[B53] Miller E. K. Freedman D. J. Wallis J. D. (2002). The prefrontal cortex: Categories, concepts and cognition. *Philos Trans R. Soc. Lond. B Biol. Sci*. 357, 1123–1136. 10.1098/rstb.2002.1099 12217179 PMC1693009

[B54] Ngo C. T. Alm K. H. Metoki A. Hampton W. Riggins T. Newcombe N. S. et al. (2017). White matter structural connectivity and episodic memory in early childhood. *Dev. Cogn. Neurosci*. 28, 41–53. 10.1016/j.dcn.2017.11.001 29175538 PMC5909412

[B55] Nomura E. M. Reber P. J. (2008). A review of medial temporal lobe and caudate contributions to visual category learning. *Neurosci. Biobehav. Rev*. 32, 279–291. 10.1016/j.neubiorev.2007.07.006 17868867

[B56] Norman K. A. O’Reilly R. C. (2003). Modeling hippocampal and neocortical contributions to recognition memory: A complementary-learning-systems approach. *Psychol. Rev*. 110, 611–646. 10.1037/0033-295X.110.4.611 14599236

[B57] Nosofsky R. M. (1986). Attention, similarity, and the identification-categorization relationship. *J. Exp. Psychol. Gen*. 115, 39–61. 10.1037//0096-3445.115.1.39 2937873

[B58] Nosofsky R. M. Palmeri T. J. (1998). A rule-plus-exception model for classifying objects in continuous-dimension spaces. *Psychon. Bull. Rev.* 5, 345–369. 10.3758/BF03208813

[B59] Nosofsky R. M. Palmeri T. J. McKinley S. C. (1994). Rule-plus-exception model of classification learning. *Psychol. Rev*. 101, 53–79. 10.1037/0033-295x.101.1.53 8121960

[B60] Nugiel T. Alm K. H. Olson I. R. (2016). Individual differences in white matter microstructure predict semantic control. *Cogn. Affect Behav. Neurosci*. 16, 1003–1016. 10.3758/s13415-016-0448-x 27444936 PMC5154852

[B61] O’Reilly R. C. Rudy J. W. (2001). Conjunctive representations in learning and memory: Principles of cortical and hippocampal function. *Psychol. Rev*. 108, 311–345. 10.1037/0033-295x.108.2.311 11381832

[B62] Palmeri T. J. Nosofsky R. M. (1995). Recognition memory for exceptions to the category rule. *J. Exp. Psychol. Learn. Mem. Cogn*. 21, 548–568. 10.1037//0278-7393.21.3.548 7602261

[B63] Park B. S. Lee D. A. Lee H. J. Kim J. Ko J. Lee W. H. et al. (2024). Correlation of diffusion tensor tractography with obstructive sleep apnea severity. *Brain Behav*. 14:e3541. 10.1002/brb3.3541 38773829 PMC11109523

[B64] Poldrack R. A. Clark J. Paré-Blagoev E. J. Shohamy D. Creso Moyano J. Myers C. et al. (2001). Interactive memory systems in the human brain. *Nature* 414, 546–550. 10.1038/35107080 11734855

[B65] Postans M. Hodgetts C. J. Mundy M. E. Jones D. K. Lawrence A. D. Graham K. S. (2014). Interindividual variation in fornix microstructure and macrostructure is related to visual discrimination accuracy for scenes but not faces. *J. Neurosci*. 34, 12121–12126. 10.1523/JNEUROSCI.0026-14.2014 25186756 PMC4152609

[B66] Ralph M. A. Jefferies E. Patterson K. Rogers T. T. (2017). The neural and computational bases of semantic cognition. *Nat. Rev. Neurosci*. 18, 42–55. 10.1038/nrn.2016.150 27881854

[B67] Ramnani N. (2006). The primate cortico-cerebellar system: Anatomy and function. *Nat. Rev. Neurosci*. 7, 511–522. 10.1038/nrn1953 16791141

[B68] Rehder B. Hoffman A. B. (2005). Eyetracking and selective attention in category learning. *Cogn. Psychol*. 51, 1–41. 10.1016/j.cogpsych.2004.11.001 16039934

[B69] Richler J. J. Palmeri T. J. (2014). Visual category learning. *Wiley Interdiscip Rev. Cogn. Sci*. 5, 75–94. 10.1002/wcs.1268 26304297

[B70] Sampaio-Baptista C. Khrapitchev A. A. Foxley S. Schlagheck T. Scholz J. Jbabdi S. et al. (2013). Motor skill learning induces changes in white matter microstructure and myelination. *J. Neurosci*. 33, 19499–19503. 10.1523/JNEUROSCI.3048-13.2013 24336716 PMC3858622

[B71] Schapiro A. C. Turk-Browne N. B. Botvinick M. M. Norman K. A. (2017). Complementary learning systems within the hippocampus: A neural network modelling approach to reconciling episodic memory with statistical learning. *Philos Trans R. Soc. Lond B Biol. Sci*. 372:20160049. 10.1098/rstb.2016.0049 27872368 PMC5124075

[B72] Schlichting M. L. Preston A. R. (2015). Memory integration: Neural mechanisms and implications for behavior. *Curr. Opin. Behav. Sci*. 1, 1–8. 10.1016/j.cobeha.2014.07.005 25750931 PMC4346341

[B73] Schlichting M. L. Preston A. R. (2017). “The hippocampus and memory integration: Building knowledge to navigate future decisions,” in *The Hippocampus from Cells to Systems: Structure, Connectivity, and Functional Contributions to Memory and Flexible Cognition*, eds Hannula D. E. Duff M. C. (Cham: Springer International Publishing), 405–437. 10.1007/978-3-319-50406-3_13

[B74] Schlichting M. L. Gumus M. Zhu T. Mack M. L. (2021). The structure of hippocampal circuitry relates to rapid category learning in humans. *Hippocampus* 31, 1179–1190. 10.1002/hipo.23382 34379847

[B75] Scholz J. Tomassini V. Johansen-Berg H. (2009). “Chapter 11—individual differences in white matter microstructure in the healthy brain,” in *Diffusion MRI*, eds Johansen-Berg H. Behrens T. E. J. (London: Academic Press), 237–249. 10.1016/B978-0-12-374709-9.00011-0

[B76] Schulte T. Müller-Oehring E. M. (2010). Contribution of callosal connections to the interhemispheric integration of visuomotor and cognitive processes. *Neuropsychol. Rev*. 20, 174–190. 10.1007/s11065-010-9130-1 20411431 PMC3442602

[B77] Seger C. A. Miller E. K. (2010). Category learning in the brain. *Annu. Rev. Neurosci*. 33, 203–219. 10.1146/annurev.neuro.051508.135546 20572771 PMC3709834

[B78] Shepard R. N. Hovland C. I. Jenkins H. M. (1961). Learning and memorization of classifications. *Psychol. Monogr. Gen. Appl.* 75, 1–42. 10.1037/h0093825

[B79] Sorg S. F. Merritt V. C. Clark A. L. Werhane M. L. Holiday K. A. Schiehser D. M. et al. (2021). Elevated intraindividual variability in executive functions and associations with white matter microstructure in veterans with mild traumatic brain injury. *J. Int. Neuropsychol. Soc*. 27, 305–314. 10.1017/S1355617720000879 32967755 PMC8462939

[B80] Stoodley C. J. (2012). The cerebellum and cognition: Evidence from functional imaging studies. *Cerebellum* 11, 352–365. 10.1007/s12311-011-0260-7 21373864

[B81] Swaminathan S. K. Freedman D. J. (2012). Preferential encoding of visual categories in parietal cortex compared with prefrontal cortex. *Nat. Neurosci*. 15, 315–320. 10.1038/nn.3016 22246435 PMC3293250

[B82] Swick D. Turken A. U. (2002). Dissociation between conflict detection and error monitoring in the human anterior cingulate cortex. *Proc. Natl. Acad. Sci. U. S. A*. 99, 16354–16359. 10.1073/pnas.252521499 12456882 PMC138615

[B83] Timmann D. Drepper J. Frings M. Maschke M. Richter S. Gerwig M. et al. (2010). The human cerebellum contributes to motor, emotional and cognitive associative learning. A review. *Cortex* 46, 845–857. 10.1016/j.cortex.2009.06.009 19665115

[B84] Wakana S. Jiang H. Nagae-Poetscher L. M. van Zijl P. C. Mori S. (2004). Fiber tract-based atlas of human white matter anatomy. *Radiology* 230, 77–87. 10.1148/radiol.2301021640 14645885

[B85] Wutz A. Loonis R. Roy J. E. Donoghue J. A. Miller E. K. (2018). Different levels of category abstraction by different dynamics in different prefrontal areas. *Neuron* 97, 716e–726e. 10.1016/j.neuron.2018.01.009 29395915 PMC6091891

[B86] Yassa M. A. Stark C. E. (2011). Pattern separation in the hippocampus. *Trends Neurosci*. 34, 515–525. 10.1016/j.tins.2011.06.006 21788086 PMC3183227

[B87] Yeh F. C. Badre D. Verstynen T. (2016). Connectometry: A statistical approach harnessing the analytical potential of the local connectome. *Neuroimage* 125, 162–171. 10.1016/j.neuroimage.2015.10.053 26499808

[B88] Yeh F. C. Liu L. Hitchens T. K. Wu Y. L. (2017). Mapping immune cell infiltration using restricted diffusion MRI. *Magn. Reson. Med*. 77, 603–612. 10.1002/mrm.26143 26843524 PMC8052951

[B89] Yeh F. C. Panesar S. Barrios J. Fernandes D. Abhinav K. Meola A. et al. (2019). Automatic removal of false connections in diffusion MRI tractography using topology-informed pruning (TIP). *Neurotherapeutics* 16, 52–58. 10.1007/s13311-018-0663-y 30218214 PMC6361061

[B90] Yeh F. C. Panesar S. Fernandes D. Meola A. Yoshino M. Fernandez-Miranda J. C. et al. (2018). Population-averaged atlas of the macroscale human structural connectome and its network topology. *Neuroimage* 178, 57–68. 10.1016/j.neuroimage.2018.05.027 29758339 PMC6921501

[B91] Yeh F. C. Verstynen T. D. Wang Y. Fernández-Miranda J. C. Tseng W. Y. (2013). Deterministic diffusion fiber tracking improved by quantitative anisotropy. *PLoS One* 8:e80713. 10.1371/journal.pone.0080713 24348913 PMC3858183

[B92] Yeh F. C. Wedeen V. J. Tseng W. Y. (2010). Generalized q-sampling imaging. *IEEE Trans Med. Imaging* 29, 1626–1635. 10.1109/TMI.2010.2045126 20304721

[B93] Zeithamova D. Bowman C. R. (2020). Generalization and the hippocampus: More than one story? *Neurobiol. Learn. Mem*. 175:107317. 10.1016/j.nlm.2020.107317 33007461 PMC7655622

[B94] Zeithamova D. Maddox W. T. (2007). The role of visuospatial and verbal working memory in perceptual category learning. *Mem. Cognit*. 35, 1380–1398. 10.3758/bf03193609 18035635

[B95] Zeithamova D. Mack M. L. Braunlich K. Davis T. Seger C. A. van Kesteren M. T. R. et al. (2019). Brain mechanisms of concept learning. *J. Neurosci*. 39, 8259–8266. 10.1523/JNEUROSCI.1166-19.2019 31619495 PMC6794919

[B96] Zhou J. Li W. Xu S. Biswal B. B. Chen H. Li J. et al. (2026). Multimodal, multifaceted, imaging-based human brain white matter atlas. *Sci. Bull*. 71, 500–504. 10.1016/j.scib.2025.08.021 40912944

