# Supplementary material for: Complementary white matter networks support flexible concept learning
Source: Front Neurosci. 2026 Sep 30;20:1877268. doi: 10.3389/fnins.2026.1877268 (PMC13662064; doi:10.3389/fnins.2026.1877268)
Supplement: Supplementary file 1 [file Table_1.pdf]

Supplementary Files for
Complementary White Matter Networks Support Flexible Concept Learning

Melisa Gumus[1,2]; Nahal Alizadeh Saghati[1]; Michael L. Mack[1]

[1] Department of Psychology, University of Toronto, Toronto, Ontario, Canada

[2] Department of Psychology, University of California Berkeley, Berkeley, California, USA

Corresponding author:

Melisa Gumus, PhD

Department of Psychology, UC Berkeley

2121 Berkeley Way, Berkeley, CA 94704

melisa.gumus@berkeley.edu

This PDF file includes:

Tables S1 to S3

Table S1. Tract bundles unique to the exception network. Each bundle within the exception network is listed alongside the number of tracts within that bundle and mean tract length.

**Tract bundle**	**Number of tracts**	**Mean length (mm)**
Corpus Callosum Forceps Minor	2756	38.64
Cingulum Frontal Parietal – Left	882	39.82
Superior Thalamic Radiation – Right	573	35.59
Superior Thalamic Radiation – Left	628	33.92
Anterior Thalamic Radiation – Right	440	33.67
Anterior Thalamic Radiation – Left	161	33.61
Fornix – Right	192	33.89
Fornix – Left	559	37.53
Dentatorubrothalamic Tract – Right	397	33.33
Dentatorubrothalamic Tract – Left	131	33.79
Superior Longitudinal Fasciculus I – Left	484	40.33
Inferior Frontal Occipital Fasciculus – Left	142	34.33
Anterior Corticostriatal Tract – Left	184	36.66
Cingulum Frontal Parahippocampal - Left	139	38.03
Reticular Tract – Right	156	33.68

Table S2. Tract bundles unique to the rule follower network. Each bundle within the rule follower network is listed alongside the number of tracts within that bundle and mean tract length.

**Tract bundle**	**Number of tracts**	**Mean length (mm)**
Middle Cerebellar Peduncle	2017	34.81
Cingulum Frontal Parietal – Right	1518	38.12
Cingulum Parolfactory – Right	810	40.55
Cingulum Parahippocampal Parietal – Right	229	32.54
Anterior Commissure	256	33.31
Inferior Longitudinal Fasciculus - Left	250	33.52
Middle Longitudinal Fasciculus – Right	226	33.43
Middle Longitudinal Fasciculus – Left	231	34.25
Cerebellum – Right	658	34.05
Cerebellum – Left	220	33.64

Table S3. Tract bundles that are overlapping between the exception and rule follower networks. Each bundle within the overlapping network is listed with the number of tracts that contributed to the network as well as the mean tract length.

**Tract bundle**	**Number of tracts**	**Mean length (mm)**
Corpus Callosum Body	4768	33.30
Corpus Callosum Tapetum	1827	34.72
Superior Longitudinal Fasciculus I – Right	447	33.69
